# Molecular Mechanisms Underlying Obesity-Induced Hypothalamic Inflammation and Insulin Resistance: Pivotal Role of Resistin/TLR4 Pathways

**DOI:** 10.3389/fendo.2019.00140

**Published:** 2019-03-08

**Authors:** Yacir Benomar, Mohammed Taouis

**Affiliations:** Paris-Saclay Institute of Neuroscience, NeuroPSI-UMR 9197, Molecular Neuroendocrinology of Food Intake, CNRS, University Paris-Sud, University Paris-Saclay, Orsay, France

**Keywords:** obesity, insulin resistance, inflammation, hypothalamus, resistin, TLR4

## Abstract

Low-grade inflammation and insulin resistance are among the clinical features of obesity that are thought to promote the progressive onset of type 2 diabetes. However, the underlying mechanisms linking these disorders remain not fully understood. Recent reports pointed out hypothalamic inflammation as a major step in the onset of obesity-induced insulin resistance. In light of the increasing prevalence of obesity and T2D, two worldwide public health concerns, deciphering mechanisms implicated in hypothalamic inflammation constitutes a major challenge in the field of insulin-resistance/obesity. Several clinical and experimental studies have identified resistin as a key hormone linking insulin-resistance to obesity, notably through the activation of Toll Like Receptor (TLR) 4 signaling pathways. In this review, we present an overview of the molecular mechanisms underlying obesity-induced hypothalamic inflammation and insulin resistance with peculiar focus on the role of resistin/TLR4 signaling pathway.

## Introduction

Obesity is a global epidemic triggering significant morbidity and mortality rate, through mainly interactions between genetic and environmental factors, notably sedentary lifestyle and unhealthy eating habits (1–3). Obesity is directly or indirectly associated to myriad of metabolic disorders and dysfunctions including chronic low-grade inflammation and insulin resistance, which are causally related to the development and progression of type 2 diabetes (T2D) (4–6). Numerous studies have been dedicated to understand the relationship between obesity, inflammation, and insulin resistance. In rodents, the consumption of High Fat Diet (HFD) predisposes to obesity, insulin resistance, and low-grade inflammation (7–9). HFD consumption altered both leptin and insulin hypothalamic responsiveness leading to the deregulation of energy homeostasis control. Indeed, these two hormones are anorexigenic and considered as key regulators of energy homeostasis (10, 11). Additionally, HFD deregulates hypothalamic neuronal circuitries, known to finely adapt hypothalamic response to body energy needs, leading to body weight gain, obesity, and T2D (12–14). More recently, hypothalamic inflammation has been identified as a critical event initiating the onset of obesity-induced insulin resistance and inflammation (4, 7, 15). Indeed, in contrast to HFD-induced peripheral inflammation, that is considered as a long-term consequence, hypothalamic inflammation acutely develops within few days of HFD consumption especially in the hypothalamic arcuate nucleus (ARC) in association with both insulin/leptin resistance and the upregulation of neuronal injury markers (8, 15, 16). These data suggest that hypothalamic inflammation is a major step in the early onset of the deregulation of energy homeostasis control and insulin resistance induced by HFD. However, the mediators and the signaling pathways triggering the onset of hypothalamic inflammation and insulin resistance are not fully characterized.

In rodents, it is largely documented that obesity alters secretory adipose tissue functions mainly adipokines and pro-inflammatory cytokines secretions (6, 17). Among these adipokines, Resistin is described as a determinant factor in obesity-mediated inflammation and insulin resistance at both central and peripheral levels (18, 19). Resistin initiates its effects through the binding to TLR4 activating pro-inflammatory signaling pathways (19–24). Moreover, TLR4 known as a component of immune system Pattern-recognition receptors (PRRs), plays a crucial role as a trigger of metabolic inflammation and insulin resistance during obesity (25–27). This review highlights molecular mechanisms underlying obesity associated hypothalamic inflammation and insulin resistance with particular focus on the role of resistin/TLR4 signaling pathway.

## Hypothalamic Control of Energy Homeostasis: Key Role of Insulin and Leptin

The hypothalamus is the main brain area controlling feeding behavior and energy homeostasis implicating complex neuronal circuits that project toward several brain regions and brainstem (28, 29). Hypothalamic arcuate nucleus (ARC), ventromedial (VMH), dorsomedial (DMH), and paraventricular (PVN) nuclei are critical for energy homeostasis control. The ARC, which sits abutting the median eminence and the third ventricle in the mediobasal hypothalamus (MBH), constitutes the key hypothalamic area that integrates peripheral hormonal and nutritional metabolic signals (10, 11, 28–32). The ARC contains two distinct functionally antagonistic neuronal populations, the orexigenic neurons expressing the agouti-related peptide (AgRP) and the neuropeptide Y (NPY) and the anorexic neurons that include cocaine-and amphetamine-regulated transcript (CART) neurons and pro-opiomelanocortin (POMC) neurons. These ARC neurons coordinate neuronal networks involved in feeding behavior and energy expenditure control (31, 32).

Among the peripheral signals, the anorexigenic effect of insulin in the hypothalamus is largely documented where insulin modulates food intake and glucose homeostasis (10, 11). Insulin crosses blood-brain barrier (BBB) in a receptor-dependent manner to reach the hypothalamus (32). In the hypothalamus, insulin receptors (IR) are highly expressed in POMC/CART and NPY/AgRP neurons (30, 31), and central delivery of insulin increases hypothalamic expression of CART and αMSH (α-melanocyte stimulating hormone), and inhibits NPY and AgRP gene expression (33, 34) reducing then food intake and body weight (35). Moreover, insulin regulates electrical activity of both POMC/CART and NPY/AgRP neurons through the stimulation of ATP-sensitive potassium (KATP) channel leading to membrane hyperpolarization and decreased firing of these neurons in a PI3K/Akt-dependent manner (35, 36). It has been also reported that insulin, through its action on hypothalamic ARC neurons, regulates hepatic glucose production (37, 38), glycogen synthesis (39), and fat metabolism (40, 41) via autonomous nervous system connections. Insulin action on AgRP/NPY neurons, suppresses hepatic glucose production (37), whereas it action on POMC neurons inhibits adipose-tissue lipolysis (41). Beside insulin, the adipose tissue-derived hormone Leptin also plays a critical role in the hypothalamic control of energy and glucose homeostasis. Leptin acts through its hypothalamic long isoform receptor (ObRb) to reduce food intake and increase energy expenditure by upregulating the expression of POMC and reducing the expression of NPY and AgRP (42–44). Leptin action within the hypothalamus also improved glucose utilization and insulin sensitivity in adipose tissue, muscle and liver (45, 46). Of note, leptin and insulin share several signaling pathways in the hypothalamus and act synergistically to modulate the central regulation of feeding and whole body energy homeostasis (47, 48).

## Impaired Hypothalamic Insulin Signaling in Obesity

In rodents, HFD consumption is considered as an important nutritional factors predisposing to obesity-induced insulin resistance. HFD consumption alters hypothalamic insulin responsiveness deregulating energy homeostasis control (7–9). Several mechanisms have been proposed to explain the loss of hypothalamic insulin action induced by HFD. Indeed, it has been shown that the impairment of hypothalamic insulin action could result from impaired transport across the BBB reducing then hypothalamic insulin uptake (49–51). Nonetheless, the direct delivery of insulin in the brain did not reverse the obesity-induced metabolic disorders (52–55) suggesting that the defect in insulin uptake into the brain is not the only mechanism. In obese animals, hypothalamic insulin resistance might be also a consequence of impaired hypothalamic insulin signaling. This could be attributed to HFD-induced hypothalamic upregulation of suppressor of cytokine signaling (SOCS3), protein tyrosine phosphatase-1B (PTP-1B) and protein kinase C, shown to blunt hypothalamic insulin signaling pathways (56–58). It is also suggested that inhibitory interplay between leptin and insulin signaling in the hypothalamus could have a causal role in the onset and the progression of hypothalamic insulin resistance. Interestingly, numerous studies clearly reported that chronic exposure to high leptin levels, which mimics obesity-associated hyperleptinemia, promotes hypothalamic insulin resistance through the impairment of neuronal insulin signaling (48, 59, 60). Furthermore, obesity-induced hypothalamic inflammation may also contribute to the development of hypothalamic insulin resistance. Indeed recent studies reported that HFD activates hypothalamic inflammatory pathways, notably NF-kB and c-jun N-terminal kinase (JNK), and increases proinflammatory cytokines leading to hypothalamic insulin resistance (4, 7, 15). In humans, increasing evidence has corroborated findings obtained from rodent models that link hypothalamic inflammation and insulin resistance to the deregulation of energy homeostasis promoting obesity onset in humans (7, 16, 61).

## Hypothalamic Inflammation and Insulin Resistance in Obesity

Epidemiological studies in obese and insulin resistant subjects have revealed a causal link between increased pro-inflammatory markers and decreased insulin sensitivity (7, 62). In rodents it is now recognized that HFD-induced metabolic inflammation contributes to metabolic disorders including insulin resistance (7, 63). This inflammatory state occurs particularly in adipose tissue, implicating the recruitment of immune cells and the activation of resident macrophages exacerbating adipose tissue inflammation and production of proinflammatory cytokines (6). These events alter insulin responsiveness of liver, skeletal muscle and adipose tissue exacerbating whole body insulin resistance (62, 63). Inflammatory processes have also been described in the brain in the context of diet induced obesity. HFD triggers local immune responses in the MBH resulting in the production of proinflammatory cytokines and neuronal injury affecting energy homeostasis control and hypothalamic insulin sensitivity (4, 4, 15, 16, 61, 64, 65). Accordingly, recent studies reported that genetic disruption of key inflammatory pathways in the hypothalamus, such as TLR4/MyD88 or IKKb/NF-kB pathways, is protective against the metabolic complications induced by HFD including hypothalamic insulin resistance (64, 66, 67). This brings further arguments for the role of hypothalamic inflammation as an important contributor of HFD-associated hypothalamic insulin resistance.

## Molecular Basis of Hypothalamic Inflammation and Insulin Resistance

At the molecular levels, IKKβ/NF-kB inflammatory pathways have been described to be critical in the development and progression of hypothalamic insulin resistance. Indeed, inactivation of IKKβ/NF-kB signaling in neurons, astrocytes or microglia of the MBH protects against HFD-induced obesity, glucose intolerance and hypothalamic insulin resistance (66, 68–70). Moreover, elevated NF-kB signaling in the hypothalamus of HFD rodents, triggers endoplasmic reticulum (RE) stress which promotes hypothalamic insulin resistance leading to the acceleration of obesity and T2D disease progression (66, 71–75). The activation of NF-kB signaling also induces hypothalamic expression of SOCS3 which impairs insulin-dependent phosphorylation of Insulin receptor and its downstream signaling (66, 68, 76). SOCS3 also targets IRS1/2 for proteasomal degradation (76). Hence, NF-kB-dependent upregulation of SOCS3 could thus link hypothalamic inflammation to insulin resistance. Like SOCS3, the PTP-1B is another signaling protein involved in the negative regulation of insulin signaling. HFD up-regulates hypothalamic PTP-1B inhibiting then insulin-mediated anorexigenic effects by insulin receptor dephosphorylation (56, 77–80). Interestingly, neuron-specific deletion of PTP-1B in mice enhanced hypothalamic insulin sensitivity and prevents HFD-induced obesity and related metabolic dysfunctions (81–83). PTP-1B deficiency also attenuates HFD-induced hypothalamic inflammation suggesting a functional link between inflammatory pathways and PTP-1B activation at the neuronal level (56, 80).

JNK signaling is another pathway proposed to be critical for the development of obesity associated hypothalamic insulin resistance. Obesity-dependent activation of JNK signaling was shown to impair insulin signaling (84). JNK promotes the serine phosphorylation of IRS-1 that inhibits insulin-dependent IRS-1 tyrosine phosphorylation and downstream signaling (85, 86). JNK deficiency in the brain protects against HFD-induced insulin resistance (87). Moreover, central administration of JNK inhibitors restores hypothalamic insulin signaling and improves impaired glucose homeostasis under HFD conditions (88).

ER stress and the unfolded protein response (UPR) also contribute to HFD-induced hypothalamic inflammation and insulin resistance (89–92). ER stress results from the deficit in protein folding capacity, and increased accumulation of unfolded protein in the ER lumen (91, 92). Prolonged ER stress leads to the activation of UPR signaling pathways in order to restore ER homeostasis (91, 92). The deleterious effects of ER stress and UPR on metabolic regulation in peripheral tissues (75, 89, 93–95) as well as in the hypothalamus (66, 90, 96) are well-documented. Recent evidence reported that IKKβ/NF-κB and JNK pathway link UPR/ER stress to obesity-induced inflammation and insulin resistance (7, 64, 95). Accordingly, the activation of IKKβ/NF-κB in the MBH was reported to increase ER stress and related metabolic disorders including insulin resistance (89, 90, 94). Conversely, neuron-specific deletion of IKKb in mice is associated with reduced UPR signaling (66). These observations support the critical role of the ER stress and UPR pathway in the onset of metabolic inflammation and insulin resistance notably in the hypothalamus.

Inflammatory responses induced by HFD are also mediated by TLRs known to be involved in innate immunity (25, 97). At the molecular levels, TLRs stimulation results in the activation of several signaling pathways such as Ikkb/NFkB, JNK/AP-1 signaling, and MAP kinases, including ERK1/2, JNK, and p38, promoting then the expression of proinflammatory cytokines notably IL1β, IL6, and TNFα (25, 97–99). Among the different members of the TLR family, TLR4 is considered as a major contributor of obesity-induced inflammation and insulin resistance (26, 27, 100). TLR4 expression is increased in obese mice and humans and positively correlates with insulin resistance (26). In obesity, TLR4 is considered as the main target for saturated fatty acids in the hypothalamus and peripheral tissues that triggers inflammatory response and endoplasmic reticulum stress promoting whole body insulin resistance (15). Recently, it has been shown that Fetuin A, a glycoprotein mainly produced by liver and adipose tissue, is required for FFAs-dependent activation of TLR4. It acts as an endogenous ligand for TLR4 that bridges FFAs and TLR4 promoting inflammation and insulin resistance (101). The implication of TLR4 in obesity was further evidenced by studies reporting that TLR4 knockdown or its pharmacological inhibition, protect mice from diet induced inflammation and insulin resistance (102–105). Furthermore, brain specific deletion of MyD88 (myeloid differentiation factor), a downstream mediator of TLR4 signaling, in mice also prevent HFD-induced obesity and associated leptin and insulin resistance (67). Accordingly, recent evidence reported that the knockdown of TLR4 in the arcuate nucleus protects rats from diet-induced weight gain, glucose intolerance and peripheral insulin resistance (106). Additionally, it has been shown that TLR4-mediated microglia signaling pathway is critical for the control of ARC neuronal activity and feeding behavior (107). Collectively, these data strongly demonstrate the implication of brain TLR4 signaling in the pathogenesis of obesity, inflammation and insulin resistance. Nevertheless, some reports do not support such a role of TLR4 in obesity and associated metabolic dysfunctions (108, 109). These discrepancies can arise from the animal models and the composition of HFD used, as well as from experimental conditions (i.e., animal age, time of exposure to HFD etc…). These contradictory data also highlight that, besides TLR4 signaling, other pathways are critically involved in obesity-associated inflammation and insulin resistance.

## Role of Central Resistin TLR4 Signaling Pathway in the Development of Hypothalamic Inflammation and Insulin Resistance

Even though the mechanisms underlying obesity-induced hypothalamic inflammation are still not fully characterized, it is now recognized that HFD promotes both central and peripheral inflammation through the increase of circulating levels of deleterious adipokines and proinflammatory cytokines originated from the adipose tissue (4–6, 17). Among these adipokines, resistin is described as a hormone linking obesity to type 2 diabetes (110, 111). Resistin is mainly secreted from adipose tissue in rodents and macrophages in humans (112, 113). Circulating levels of resistin positively correlate with obesity in rodents, promoting both inflammation and insulin resistance (114–117). In rodents, peripheral overexposure to resistin impairs insulin responsiveness (118–120). Conversely, loss of resistin or infusion of resistin antibodies improves peripheral insulin sensitivity (17, 121–123). Although resistin has been conclusively linked to the development of obesity and insulin resistance in rodents, disagreement persists regarding the pathogenic role of resistin in human obesity. Several studies support the positive relationship between insulin resistance and elevated plasma resistin levels in obese and type 2 diabetic individuals (124–126), whereas other studies have shown contradictory findings (127, 128). Beside its role in obesity and insulin resistance, resistin is greatly implicated in proinflammatory processes which are causally involved in the development of insulin resistance in both rodents and humans (110, 111, 129–131). Resistin regulates the production of key pro-inflammatory cytokines, such as TNFα and IL6, through the activation of NF-κB signaling pathways in macrophages contributing to profound alterations of peripheral insulin signaling pathways resulting in an insulin-resistant state (17, 110, 129, 130, 132, 133). Previous studies showed that resistin is also expressed in the hypothalamus (134). Interestingly, central resistin activates hypothalamic neurons, and modulates food intake, glucose homeostasis and lipid metabolism in addition to the alteration of liver insulin sensitivity (135–138), suggesting an important role of hypothalamic resistin on the control of energy homeostasis and peripheral insulin sensitivity. Nevertheless, the role of resistin on the development of brain inflammation and insulin resistance remains poorly documented. At the molecular level, the resistin receptor remains uncharacterized. However, recent investigations reported TLR4 as a potential candidate for human resistin. TLR4 was first reported to mediate the proinflammatory effects of resistin in human myeloid and epithelial cells (19). TLR4 was also reported to mediate resistin effects on breast cancer progression (139). Recently, it has been shown that resistin competes with LPS for binding to TLR4 resulting in the inhibition of LPS-induced proinflammatory responses and the upregulation of anti-inflammatory pathways suggesting a protective role for resistin/TLR4 pathway against endotoxic shock (24). More recently, we reported that resistin acts within the hypothalamus through the activation of TLR4 receptor and its downstream signaling, promoting both hypothalamic and peripheral inflammation, leading consequently to the impairment of insulin, adiponectin and FGF21 signaling in the hypothalamus and peripheral insulin-sensitive tissues (20, 21).

## By which Mechanisms Resistin Promotes Hypothalamic Insulin Resistance (Figure 1)?

Resistin directly binds to TLR4 in the hypothalamus, promoting the recruitment of the adaptors proteins MyD88 and TIRAP and the activation of downstream signaling pathways. The activation of Resistin/TLR4 signaling impairs hypothalamic insulin responsiveness through the alteration of the insulin-mediated insulin receptor (IR), AKT, and ERK1/2 phosphorylations. This could be attributed to the resistin-dependent downregulation of IR as well as the upregulation of the hypothalamic expression of SOCS-3 and PTP1B, known as key negative modulators of insulin signaling (56, 76). Resistin-dependent upregulation of the serine phosphorylation of IRS-1 may also contribute to the impairment of hypothalamic insulin signaling through the activation of the serine kinases JNK and p38 MAPK), which are reported to promote the serine phosphorylation of IRS-1 leading to insulin resistance (85, 86, 140). Moreover, Resistin triggers hypothalamic inflammation as evidenced by the upregulation of the hypothalamic expression of pro-inflammatory cytokines such as IL6, and the activation of JNK and p38 MAPK signaling pathways known to promote inflammatory response (20). It is noteworthy that HFD-induced activation of microglia and astrocytes is critically involved in the development of hypothalamic inflammation and associated metabolic dysfunctions including insulin resistance (16, 69, 141–143). This leads to reactive gliosis evidenced by the proliferation and recruitment of activated astrocytes and microglia in the MBH amplifying hypothalamic inflammatory response (16, 144–146). Since resistin has strong effect on hypothalamic inflammation, similar mechanism might be suspected.

**Figure 1:**
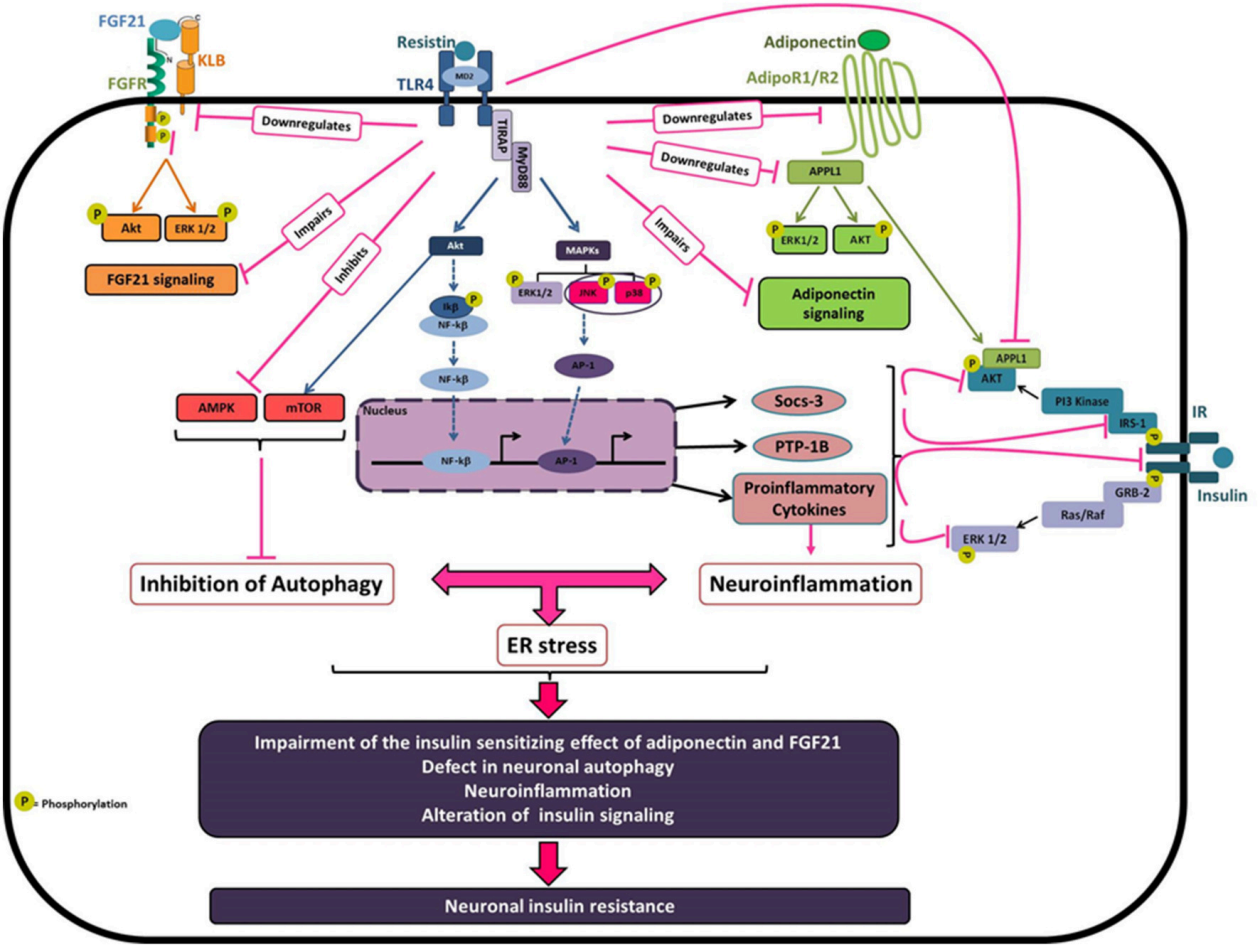
Molecular basis of resistin-dependent neuronal insulin resistance. Obesity is associated with elevated circulating resistin which may reach the hypothalamus leading to hypothalamic inflammation and insulin resistance. Resistin binding to TLR4 result in recruitment of two adaptor-associated proteins TIRAP and MyD88 that leads to the activation of different downstream signaling pathways especially Akt, ERK1/2, JNK, and p38MAPK. Once triggered, Resistin/TLR4 signaling decreases both IR and IRS expression and activity by the downregulation in tyrosine phosphorylation. In agreement with that, resistin upregulates the negative regulators of insulin signaling SOCS-3, and PTP-1B, and activates the MAP kinases JNK and p38 which promotes the phosphorylation of IRS1/2 on serine 307 resulting in the desensitization of insulin signaling. Resistin/TLR4 signaling also promotes neuronal inflammation, at least in part via the activation of the transcription factors AP-1 and NF-kB leading to the upregulation of proinflammatory cytokines notably, IL-6, TNFα, and IL-1β, which interferes with insulin signaling promoting neuronal insulin resistance. Resistin, via TLR4, also decreases neuronal autophagy, through to the inhibition of AMPK phosphorylation and the activation of Akt/mTOR, which could promote neuronal ER stress and inflammation leading to an amplification loop. Besides, resistin-dependent neuronal insulin resistance could also be attributed to the impairment of both adiponectin and FGF21 signaling known as insulin-sensitizing hormones. Resistin decreases the expression levels of adiponectin receptors AdipoR1/R2 and promotes the downregulation of the adaptor protein APPL1, which is implicated in AdipoRs signaling contributing to the insulin-sensitizing effect of adiponectin. Additionally, resistin reduces the expression of FGF21 and its receptor components FGFR1 and KLB that contribute to the impairment of FGF21 signaling and its beneficial effects on insulin sensitivity.

Another suggested mechanism is that central resistin might induce hypothalamic insulin resistance through the impairment of adiponectin and FGF21 signaling known as insulin-sensitizing hormones (147–152). Central resistin treatment reduced the expression levels of adiponectin receptors AdipoR1/R2 in the hypothalamus of mice and rats and promotes the downregulation of the adaptor protein APPL1 (21). Indeed, APPL1 is implicated in AdipoRs signaling contributing to the insulin-sensitizing effect of adiponectin (147, 153). Resistin-dependent downregulation of APPL1 enhanced Akt association with its endogenous inhibitors TRB3 inhibiting then insulin-mediated Akt signaling (21). Additionally, central resistin treatment markedly reduced hypothalamic expression of FGF21 and its receptors FGFR1 and KLB that could promote the impairment of hypothalamic FGF21 signaling and its beneficial effects on insulin sensitivity. Accordingly, resistin overexposure directly impairs both FGF21 and adiponectin signaling in human and mouse neuronal cells. Interestingly, the knockdown of TLR4 prevents resistin-dependent impairment of adiponectin and FGF21 signaling in mice and neuronal cells suggesting a critical role of TLR4 in mediating resistin effects (21).

Other candidate for resistin downstream effects on hypothalamic insulin sensitivity could be the alteration of neuronal autophagy. In fact, the disruption of autophagy in the hypothalamic neurons is critically involved in diet-induced obesity and associated hypothalamic inflammation and insulin resistance (16, 154–158). Interestingly, in neuronal cells, resistin overexposure decreases neuronal autophagy through the repression of several autophagy markers, especially LC3, ATG7, and Beclin-1. At the molecular levels, resistin exerts its effects via the activation of TLR4 signaling leading to the inhibition of AMPK phosphorylation and the activation of Akt/mTOR which regulate autophagy (22). These results were validated in mice, where resistin treatment decreases hypothalamic expression of key autophagy markers. In particular, resistin strongly diminished LC3 labeling in the arcuate nucleus through a mechanism involving TLR4 signaling (22). All together, these data clearly reveal resistin/TLR4 as a new regulatory pathway of neuronal autophagy, and suggest that resistin-dependent neuronal autophagy could be a key contributor of hypothalamic inflammation and insulin resistance.

## Conclusion

Obesity is associated with low grade inflammation which occurs in peripheral metabolic tissues, resulting in whole body insulin resistance, but also in the hypothalamus causing local impairment of insulin signaling and sensitivity. Our recent findings evidenced that central Resistin/TLR4 signaling pathway promotes the onset of hypothalamic inflammation and insulin resistance. Despite these evidences, further studies are needed to clarify the specific role of resistin produced within the hypothalamic neurons as compared to that produced in the periphery, and to identify the neural circuitries mediating resistin effects within hypothalamic nuclei. Indeed, it is also necessary to elucidate the role of resistin/TLR-4 signaling pathway in hypothalamic astrocytes and microglia cells, which are critically involved in the onset of hypothalamic inflammation. Further investigations are needed to clearly decipher regulatory mechanisms involved in hypothalamic resistin/TLR4 signaling in the context of obesity. This will constitute a major step to understand the molecular mechanisms underlying the onset of obesity-induced neuroinflammation, and will identify new potential therapeutic targets to overcome obesity-associated hypothalamic inflammation and related metabolic disorders.

## Author Contributions

YB wrote the manuscript and produced the final version. MT contributed to the redaction and reviewed the manuscript.

### Conflict of Interest Statement

The authors declare that the research was conducted in the absence of any commercial or financial relationships that could be construed as a potential conflict of interest.
